# pH stability during fermentation is associated with sustained antibacterial metabolite production in marine sediment *Bacillus* species

**DOI:** 10.1128/aem.02595-25

**Published:** 2026-03-18

**Authors:** Kitsa C. Uzima, Trust Mambane, Abraham G. Ogofure, Ezekiel Green

**Affiliations:** 1 Department of Biotechnology and Food Technology, University of Johannesburg, Doornfontein Campus 61799, Johannesburg, South Africa; 2 Microbial Pathogenicity and Molecular Epidemiology Research Group (MPMERG), Department of Biotechnology and Food Technology, University of Johannesburg, Doornfontein Campus 61799, Johannesburg, South Africa; University of Delaware, Lewes, Delaware, USA

**Keywords:** pH stability, secondary metabolites, fermentation, antimicrobial activity, marine bacteria, *Bacillus*

## Abstract

Globally, the rise in antibiotic-resistant pathogens has underscored the urgent need for new strategies to discover antimicrobials, with emphasis on microbial producers of secondary metabolites. The influence of pH on bacterial recovery, metabolite expression, and antibacterial activity in isolates from marine sediments was evaluated in this study. Three culture media were used to isolate sediment bacteria across a pH gradient (5.0–8.0), and conventional biochemical methods were employed for putative identification of the bacterial isolates. The agar plug assay was used for primary antibacterial screening, while the disk diffusion assay of the cell-free and sonicated extracts was used for secondary screening after 7 days of submerged fermentation of the isolates at their different culture pH levels. The results revealed the predominance of *Bacillus* species from the different pH levels, with zones of inhibition ranging from 10.00 ± 1.00 to 47.50 ± 2.50 mm against clinical and environmental isolates. The four *Bacillus* species-like isolates selected for submerged fermentation showed a pH drift toward alkalinity, except for the culture initiated at pH 7.5, which remained stable. The secondary screening revealed a markedly reduced antibacterial activity for all isolates (≤9 mm) compared to primary screening, with the pH 7.5 isolate retaining the strongest inhibition. The findings suggest that pH stability during fermentation was strongly associated with sustained antibacterial activity, with isolates maintained at near-constant pH retaining significantly higher inhibitory activity than those in cultures whose pH increased during fermentation. This highlights a key consideration for the bioprospecting workflow targeting biosynthetic gene clusters and producers of secondary metabolites.

**IMPORTANCE:**

Marine environments are important reservoirs of bacteria capable of producing bioactive secondary metabolites; however, many promising antimicrobial producers identified during initial screening fail to retain activity during fermentation. This study demonstrates that pH stability during fermentation, rather than pH value alone, is a key determinant of sustained antibacterial metabolite production in marine sediment-derived *Bacillus* species. By linking isolation conditions, fermentation physiology, and bioactivity outcomes, the findings provide practical guidance for improving the reliability of marine bioprospecting and antimicrobial discovery pipelines. These insights are particularly relevant for efforts to recover stable antimicrobial producers from complex environmental systems.

## INTRODUCTION

The emerging and re-emerging antimicrobial resistance (AMR) crisis is one of the most serious threats to public health and humanity worldwide, and innovative solutions are needed to curb it. The AMR phenomenon, in which pathogens develop mechanisms to evade the effects of antimicrobials, is not only a future concern but also a stark reality with devastating consequences of global significance (1, 2). The severity of this silent pandemic is shown by reports, which estimated that 1.3 million deaths recently were due to bacterial antimicrobial resistance, and nearly 5 million deaths occurred in 2019 (2–4). Without stronger action to curb this silent menace, the estimated annual AMR-related deaths could approach 8–10 million by the year 2050 (2–4). The failure of existing, conventional antimicrobials underscores a critical need to expand the search for novel compounds beyond well-studied terrestrial sources and to explore unconventional ecosystems, such as the marine environment.

The marine ecosystem represents the largest underexplored habitat on Earth, with a diverse array of microbial communities and a unique physicochemical environment (5, 6). The unique conditions (high salinity, pressure, and limited nutrients) of the marine environment, especially in marine sediments, can force marine microbes to evolve new biochemical pathways for survival, thereby leading to the production of antimicrobial compounds with unique structures and mechanisms of action (7, 8). The metabolic pathways developed by microbial communities in marine sediments often led to the production of secondary metabolites with potent biological activities (8, 9), in contrast to those of microbes from terrestrial ecosystems. Consequently, secondary metabolites from marine-derived bacteria and actinobacteria have become a growing focus of natural product research, thereby contributing new chemical classes such as marine lipopeptides, halogenated polyketides, and brominated alkaloids (10–12). Despite interest in exploring the marine ecosystem for novel antimicrobials, research on marine sediment microbiota remains scarce, largely because isolates can be difficult to cultivate in the laboratory.

Moreover, a significant proportion of biosynthetic gene clusters (BGCs) in marine-sediment microbes might remain cryptic (silent) under standard conditions (13, 14), and targeted cultivation under varied physicochemical conditions, such as altering pH, might stimulate secondary metabolite production. Nonetheless, there is a dearth of information about how environmental pH shapes both stability during cultivation (particularly during the transition from solid-state agar-based screening to submerged liquid fermentation) and eventual metabolite expression. Notwithstanding the interest in marine sediment bacteria as sources of bioactive secondary metabolites, it remains unclear whether the antimicrobial activity detected during agar-based primary screening reliably translates to sustained antimicrobial activity under dynamic pH conditions typical of liquid cultivation (fermentation).

More specifically, the role of pH stability during liquid cultivation, rather than the initial pH, in maintaining secondary metabolite production and expression has not been systematically investigated. This study was therefore aimed at isolating antimicrobial-producing bacteria from marine sediments across a pH gradient, evaluating antibacterial activity using the agar plug and liquid extract assays, and determining the effect of fermentation-associated pH shifts on metabolite bioactivity. The findings aim to contribute to the science of optimization and cultivation strategies for marine natural product discovery and to support Sustainable Development Goals (SDGs), such as good health and well-being (SDG-3), responsible consumption and production (SDG-12), and life below water (SDG-14).

## MATERIALS AND METHODS

### Study site and sample collection

Marine sediment samples were previously collected from Sodwana Bay, South Africa, a coastal environment within the iSimangaliso Wetland Park known for its biodiverse marine ecosystem and dynamic physicochemical gradients (15). The samples were obtained aseptically from diverse locations using sterile scoops, transferred into pre-sterilized containers, and stored on ice during transport to the laboratory. Upon arrival, they were refrigerated and subsequently utilized by screening for producers of secondary metabolites.

### Bacterial isolation across a pH gradient

The presence of heterotrophic bacteria was evaluated using nutrient agar (NA), marine agar (MA), and actinomycete isolation agar (AIA) (three different culture media) to ensure the recovery of both fast- and slow-growing bacterial species. Each medium was prepared and adjusted to a range of pH values (beginning with 4.5, 5.0, 5.5, 6.0, 6.5, 7.0, 7.5, to 8.0) using 1 M HCl and 1 M NaOH, and the pH adjustments were verified using a calibrated pH meter (Lasec Bench Meter, XS Instruments, Italy). The standard plate method was employed for bacterial enumeration and isolation (16). Each pH-adjusted medium was poured into sterile Petri dishes containing 200 µL of sediment suspension, which was then inoculated onto the plates in triplicate. All the plates were appropriately labeled and incubated at 25°C for 24–120 h under ambient conditions (17). Following incubation, distinct bacterial colonies were enumerated and morphologically observed.

### Observation of plates for isolates with natural inhibitory properties

During enumeration, plates were closely examined for colonies exhibiting zones of inhibition or clearance, suggesting antagonistic interactions within the mixed microbial communities. These colonies were carefully isolated and purified (sub-cultured) onto fresh nutrient agar plates (at the pH at which they were cultivated) to obtain pure cultures. The isolates were considered potential producers of secondary metabolites and were subjected to further antibacterial screening (18).

### Primary screening for antibacterial activity (agar plug method)

Putative secondary metabolite-producing isolates were cultured on nutrient agar plates adjusted to the pH of their original isolation conditions. After 4 days of incubation at 25°C, 6-mm-diameter agar plugs were aseptically cut from the actively growing regions of the cultures using a sterile cork borer. The plugs were carefully placed onto the surface of freshly prepared nutrient agar plates that had been previously seeded with standardized bacterial test organisms (0.5 McFarland turbidity standard, corresponding to 1.5 × 10^8^ CFU/mL). The test organisms comprised eight bacterial strains, including four autochthonous isolates recovered from the same marine sediment and four clinically relevant pathogens of public health importance: *Klebsiella pneumoniae*, *Pseudomonas aeruginosa*, *Escherichia coli*, and *Staphylococcus aureus*. Following inoculation, the plates were incubated for 24 h at 37°C, after which the zones of inhibition surrounding the agar plugs were measured in millimeters as indicators of antibacterial activity (19, 20). Isolates producing visible inhibition zones were selected for secondary metabolite production and secondary screening.

### Fermentation and monitoring of pH dynamics

Based on primary screening results, four potent producer isolates with broad or strong inhibitory zones were selected for secondary metabolite production. These isolates were obtained at pH 5.0, 7.0, 7.5, and 8.0. Nutrient broth (1.4 L in 2 L Erlenmeyer flasks) was prepared and adjusted to the respective pH levels with 1 M HCl and 1 M sodium bicarbonate. Each broth medium was inoculated with 100 mL of a standardized (0.5 McFarland) culture of the selected isolates. The cultures were incubated in a rotary shaking incubator at 180 rpm and 30°C for 7 days to promote aeration and metabolite secretion. pH was measured at two time points: immediately after inoculation (day 0) and at harvest (day 7). Continuous pH monitoring or dynamic pH control during fermentation was not performed, as the objective was to observe pH drift under uncontrolled conditions representative of standard batch fermentation. Media were not buffered during fermentation. At the end of fermentation, the final pH of each broth culture was recorded. Cultures were stored at 4°C prior to metabolite extraction.

### Extraction of secondary metabolites

After fermentation, the cultures were centrifuged for 15 min at 5,000 rpm in a refrigerated (4°C) centrifuge (Thermo Fisher Scientific, Germany). The cell-free supernatant (CFS) was decanted and stored at 4°C, while the pellet fractions (cell mass) were resuspended in 20 mL of sterile distilled water, vortexed for 5 min, then sonicated (LABOTEC, South Africa) for 10 min at high intensity to release intracellular metabolites. The sonicated suspensions were re-centrifuged under identical conditions, and the resulting supernatants were retained as intracellular extract fractions. This approach allowed comparison of extracellular and intracellular metabolite activity (21).

### Secondary screening for antibacterial activity

Secondary screening was performed employing the disc diffusion technique (Kirby-Bauer method). Sterile paper discs (6 mm) were impregnated with undiluted cell-free extracts (CFE), 1:1 diluted CFE, undiluted sonicated extracts (SON), and 1:1 diluted sonicated extracts. Extracts were not pH-adjusted prior to testing; they were applied to discs at their native post-fermentation pH. Discs were placed on sterile nutrient agar plates that had been seeded with six standardized test pathogens comprising gram-positive bacteria (*Bacillus subtilis* ATCC 11774, *Enterococcus faecalis* ATCC 29212, and *Staphylococcus aureus* ATCC 12493) and gram-negative bacteria (*Pseudomonas aeruginosa* ATCC 9027, *Klebsiella pneumoniae* NCIMB 10102, and *Escherichia coli* ATCC 8739), all but one obtained from the American Type Culture Collection. The plates were incubated at 37°C for 24 h, and the zones of inhibition were measured in millimeters. All experiments were conducted in triplicate, and results were expressed as mean ± standard deviation (22). Uninoculated, pH-adjusted nutrient broth controls incubated under identical conditions showed no inhibition zones, confirming that observed activity was attributable to bacterial metabolites.

### Extraction of crude bioactive compounds

Crude extraction of secondary metabolites from the CFS was performed using ethyl acetate as the extraction solvent. Equal volumes of CFS (500 mL) and ethyl acetate (500 mL) were mixed in a separating funnel and shaken vigorously for 30 min at 30°C to ensure efficient partitioning of metabolites. The mixture was then left overnight to allow complete phase separation. The ethyl acetate layer (organic phase) was collected, concentrated under reduced pressure using a rotary evaporator (LabTech VP30, South Africa), and stored at 4°C for further analysis (22).

### Identification of bacterial isolates

All bacterial isolates, including potential metabolite producers and environmental test isolates, were characterized using conventional microbiological techniques to evaluate putative identity following standard procedures. Morphological characterization included observation of colony morphology and Gram staining. Biochemical characterization included Gram staining, KOH string test, catalase test, citrate utilization (Simmons citrate agar), and triple sugar iron (TSI) agar test, urease test, motility, and spore staining (23–25). Taxonomic assignments are putative and based solely on phenotypic characteristics, as molecular identification (e.g., 16S rRNA gene sequencing) was not performed, which represents a key limitation of this study.

### Chemical profiling (GC-MS)

GC-MS analysis (volatile profiling) was performed using an Agilent 8890 GC system coupled to a mass spectrometer (Agilent Technologies, Palo Alto, United States). The GC-MS system was equipped with an Agilent HP-5MS 5% phenyl-95% methylsiloxane column (30 m length × 0.25 mm internal diameter × 0.25 µm film thickness). The system was operated at an injection temperature of 280°C, using helium as the carrier gas at a constant flow rate of 1.4 mL/min. The oven temperature program used started from 40°C, held for 1 min, and ramped at 10°C/min to a final temperature of 300°C with a final hold time of 10 min. The mass spectrometry was operated in scan mode using the electron ionization source and a quadrupole mass analyzer, which was set up to a scan range of 50–600 m/z. Data acquisition and processing were carried out using Agilent MassHunter software, which used the NIST library for compound identification via mass spectral library searches. Compound annotation was achieved through spectral matching against the NIST mass spectral library, with a match factor threshold of ≥70%. GC-MS was used for chemical fingerprinting and compound class identification; individual metabolites were not purified or tested for bioactivity (26, 27). Full chromatograms and putative identification of some compounds are provided as supplemental material (Tables S1 and S2, as well as Fig. S3 and S4).

### Statistical analysis

Data obtained in the study were compiled in spreadsheets and analyzed using R-Studio version 4.5, employing packages *ggplot2*, *dplyr*, *tidyr*, *patchwork*, *ggtext*, *ComplexHeatmap*, *circlize*, *grid*, and *cowplot* (18, 26–28). The relationship between pH and cultivable bacterial diversity was evaluated using the *χ*^2^ test of independence at a 95% confidence level to determine whether variations in pH significantly influenced bacterial recovery and distribution across media types. Inhibition zones are reported as mean ± standard deviation from triplicate assays.

## RESULTS AND DISCUSSION

### Media performance and bacterial recovery across a pH gradient

**Fig 1:**
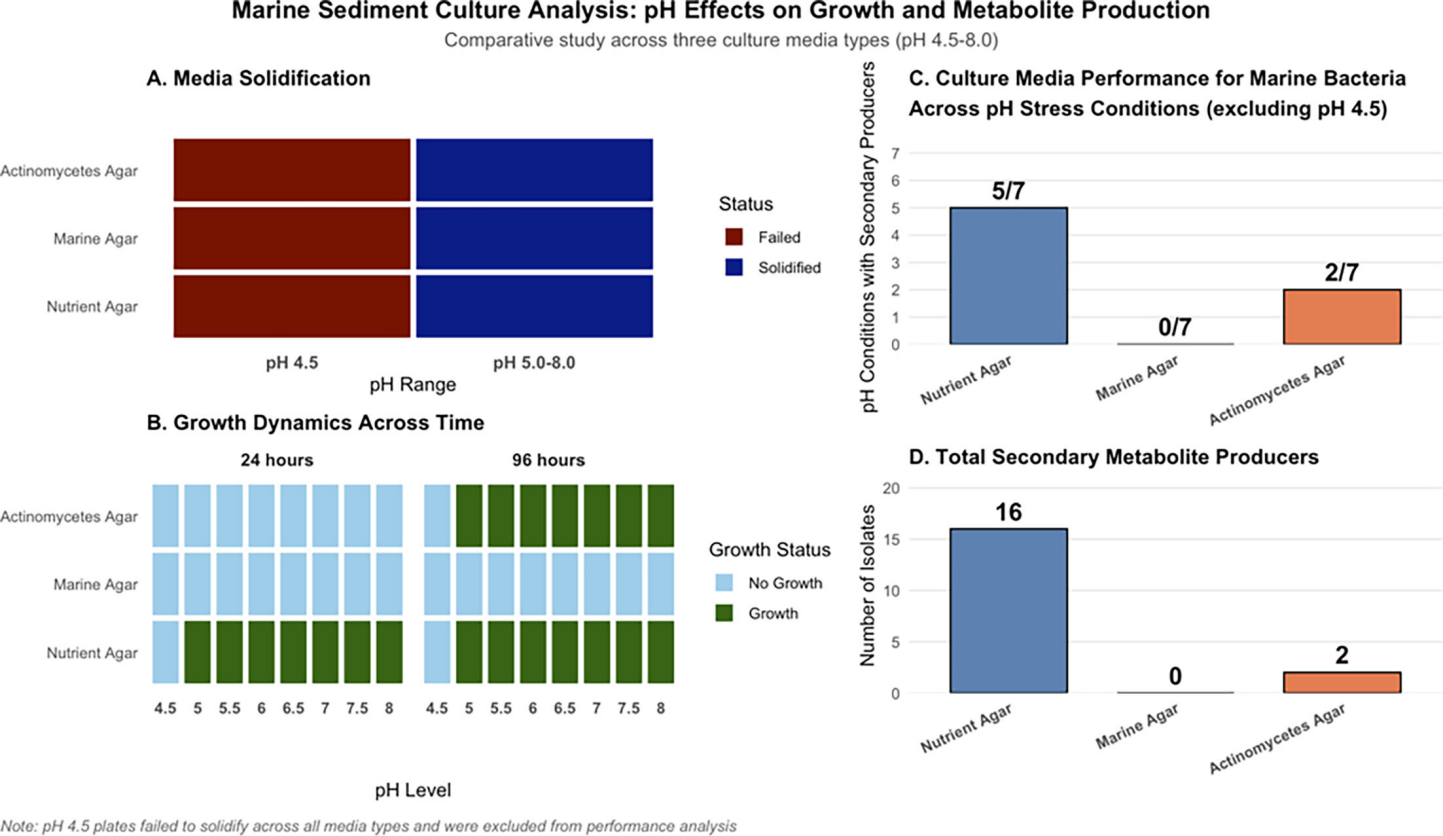
Marine sediment culture analysis showing the influence of pH on bacterial growth and secondary metabolite production across three media types. (**A**) Agar solidification performance of the different culture media across the tested pH range. (**B**) Bacterial growth patterns observed in the culture media under varying pH conditions and incubation temperatures. (**C**) Distribution of isolates producing secondary metabolites across the different pH conditions evaluated. (**D**) Total number of secondary metabolite–producing isolates recovered from each culture medium

All three media (marine, nutrient, and actinomycete isolation agars) failed to solidify at pH 4.5, likely due to acid-catalyzed agar hydrolysis. The consistency of the media at pH 5.0–6.5 was suboptimal for standard microbiological techniques, though solidification did occur. Bacterial growth occurred rapidly on nutrient agar at all pH levels except 4.5, whereas marine agar supported no visible growth at either 24 or 96 h post-incubation. Actinomycete isolation agar showed delayed growth, with colonies emerging only after 96 h, indicating its suitability for slow-growing species (Fig. 1). Nutrient agar provided the most favorable conditions for recovery of bacterial growth and metabolite producers from marine sediments, whereas AIA supported limited, slow-developing colonies, and MA proved unsuitable under the tested conditions.

**Fig 2:**
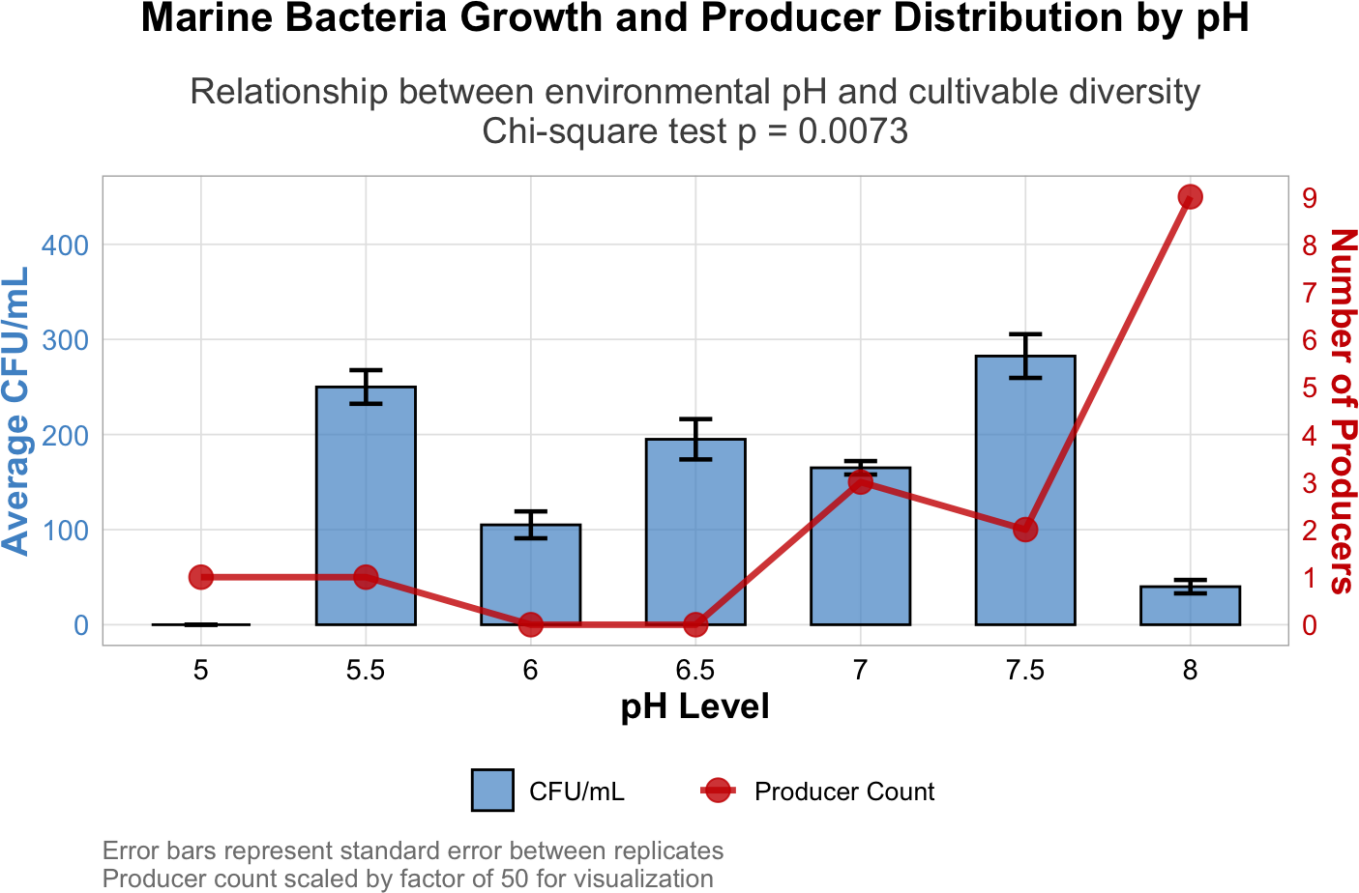
Heterotrophic bacterial count across the pH range and potential secondary metabolite producer count.

The heterotrophic bacterial count, expressed as CFU/mL, varied significantly across the pH range tested on nutrient agar (*χ*^2^ test, *P* = 0.0073). The lowest count, below 40 CFU/mL, was recorded at pH 5.0, while the highest count (>250 CFU/mL) was observed at pH 7.5, indicating that near-neutral conditions supported optimal bacterial growth. Moderate colony formation occurred between pH 5.5 and 7.0, followed by a slight decline at pH 8.0 (Fig. 2). These findings align with extensive evidence that pH is a primary environmental factor structuring microbial diversity and abundance in marine sediments (29–32).

The distribution of potential secondary metabolite producers, as indicated by inhibition zones on mixed culture plates, followed a distinct pattern. The number of producers peaked at pH 8.0 (*n* = 9), whereas no producer activity was detected at pH 6.0 and 6.5. This suggests that metabolite biosynthesis in sediment-derived bacteria is favored under slightly alkaline conditions, possibly linked to stress responses and activation of biosynthetic gene clusters (30, 33, 34).

### Putative identification of bacterial isolates

Morphological and biochemical characterization indicated that the majority of recovered isolates belonged to the genus *Bacillus*. All isolates were gram-positive rods, displaying typical marine bacterial traits. TSI agar was used as a differential carbohydrate utilization assay to assess glucose (0.1%), lactose (1%), and sucrose (1%) fermentation, as well as gas and hydrogen sulfide (H_2_S) production. For the putative *Bacillus* isolates, a consistent alkaline slant/acid butt (K/A) reaction, without gas or H_2_S production, was observed in most isolates, indicating glucose fermentation only, consistent with the metabolic profile reported for many *Bacillus* species. Occasional acid/acid (A/A) (slant/butt) reactions likely reflect limited sucrose utilization and were interpreted cautiously. No black precipitate or gas formation was detected in any isolate. More so, based on citrate utilization and additional biochemical tests, putative species-level assignments included *B. subtilis* and other *B. subtilis-*like organisms, which are known for their marine or environmental spore-forming potentials and association with bioactive metabolite production (Table 1). The dominance of *Bacillus* spp. aligns with reports from similar marine sediment environments (35–37).

**TABLE 1:** Morphological and biochemical characterization of the putative bacterial isolates^*a*^

Code	Gram stain	Spore stain	Catalase	TSI (slant/butt; gas/H_2_S)	Citrate utilization	Urease	Oxidase	Growth at 30°C–37°C	Putative taxonomic assignment^*b*^
P. 1	+	−	+	K/A; −/−	−	+	−	+	*Brevibacterium* sp.
P. 2	+	+	+	K/A; −/−	+	+	−	+	*Bacillus* sp.
P. 3	+	+	+	K/A; −/−	+	+	−	+	*Bacillus* sp.
P. 4	+	+	+	K/A; −/−	+	+	−	+	*Bacillus* sp.
P. 5	+	−	+	K/A; −/−	−	+	−	+	*Corynebacterium* sp.
P. 6	+	+	+	K/A; −/−	+	+	−	+	*Bacillus* sp.
P. 7	+	−	+	K/A; −/−	−	+	−	+	*Brevibacterium* sp.
P. 8	+	+	+	K/A; −/−	+	+	−	+	*Bacillus* sp.
P. 9	+	+	+	A/A; −/−	+	+	−	+	*Bacillus* sp.
P. 10	+	+	+	K/A; −/−	+	+	−	+	*Bacillus* sp. (subtilis-like)
P. 11	+	+	+	K/A; −/−	+	+	−	+	*Bacillus* sp. (subtilis-like)
P. 12	+	+	+	K/A; −/−	+	+	−	+	*Bacillus* sp. (licheniformis-like)
P. 13	+	+	+	K/A; −/−	+	+	−	+	*Bacillus* sp.
P. 14	+	+	+	K/A; −/−	+	+	−	+	*Bacillus* sp. (pumilus-like)
P. 15	+	+	+	K/A; −/−	+	+	−	+	*Bacillus* sp. (subtilis-like)
P. 16	+	+	+	A/A; −/−	+	+	−	+	*Bacillus* sp. (amyloliquefaciens-like)

^*a*^ P. 1 to P. 16, producer 1 to producer 16; +, positive; −, negative.

^*b*^ Putative bacterial identification based on biochemical tests. Species-level identification remains provisional and would require additional confirmatory analyses, including molecular characterization.

All isolates were gram-positive rods, positive for spore formation by endospore staining, catalase positive, urease positive, citrate positive or negative depending on isolate, and negative in the KOH string test, confirming the absence of gram-negative cell wall characteristics. Growth was observed on general nutrient agar. Based on the combined morphological and biochemical characteristics, isolates were conservatively assigned to the genus *Bacillus* or closely related genera. Species-level identification was considered putative and requires molecular confirmation using 16S rRNA gene sequencing or whole-genome analysis.

### Primary antibacterial screening: pH-dependent activity profiles

**Fig 3:**
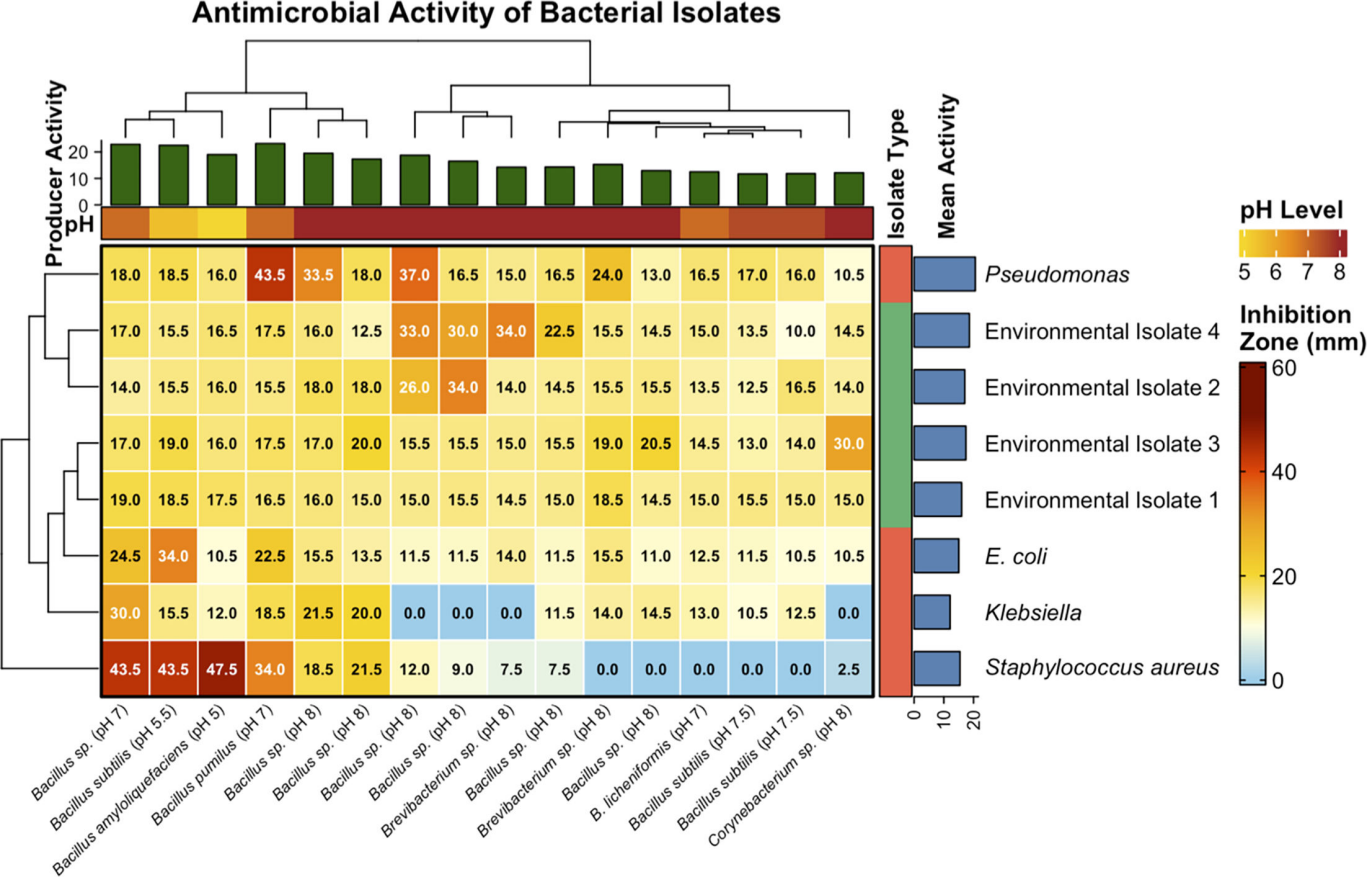
Primary screening of antibacterial activity (agar plugs) of marine sediment bacteria isolated at different pH levels.

Primary screening using the agar plug method revealed broad-spectrum antibacterial activity among the producer isolates, with zones of inhibition ranging from 10 to 47.5 mm (Fig. 3). The most pronounced activity was recorded for putative *B. subtilis* (pH 7.0−7.5) and *Bacillus* species (pH 7.5), both of which exhibited strong inhibition (>20 mm) against *Pseudomonas* and *Klebsiella* species. Moderate inhibition was observed for other isolates, while some strains showed limited or no activity against *Staphylococcus aureus*. Cluster analysis grouped isolates according to activity intensity, with pH 7.0−7.5 producers forming a distinct high-activity cluster. These findings are consistent with reports that *Bacillus* species exhibit maximal antimicrobial metabolite production around pH 7.0−8.0 (38, 39).

### Fermentation-associated pH dynamics

**Fig 4:**
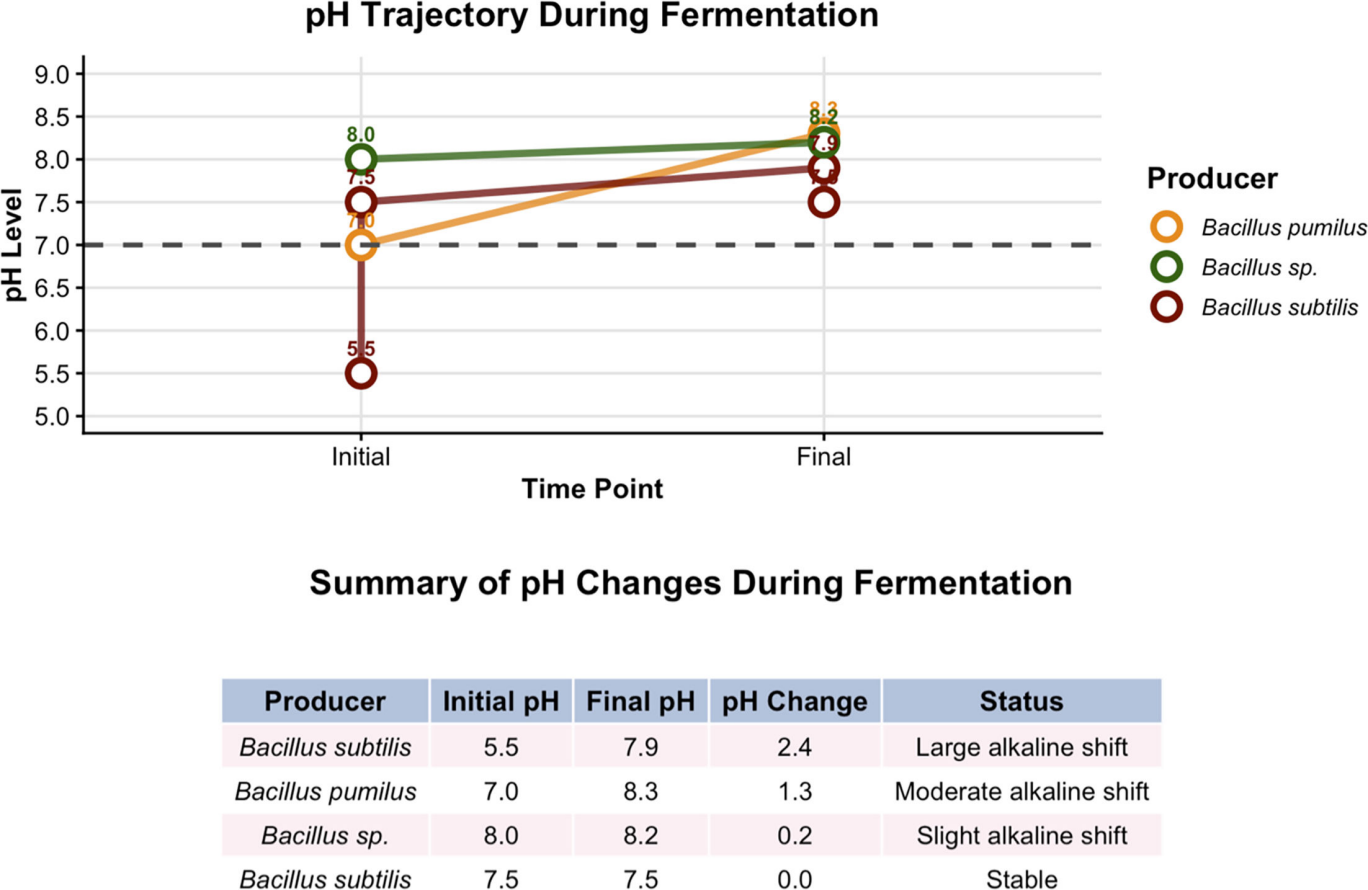
Cowplot of pH trajectory and summary of pH changes during fermentation.

All fermentation broths increased in pH between day 0 and day 7, except for the culture maintained at an initial pH of 7.5, which remained stable (ΔpH = 0.0). The largest alkaline shift was observed for putative *B. subtilis* cultured at an initial pH of 5.5, where the pH rose to 7.9 (ΔpH = +2.4), indicating substantial metabolic activity and likely the production of alkaline byproducts. Another *Bacillus* species, initiated at pH 7.0, exhibited a moderate increase to pH 8.3 (ΔpH = +1.3), while *Bacillus* sp. at pH 8.0 showed only a slight elevation to pH 8.2 (ΔpH = +0.2; Fig. 4). The stability observed at pH 7.5 suggests metabolic equilibrium under near-neutral conditions.

This pH trajectory reflects a strong link between metabolic activity and environmental alkalinization, a hallmark of *Bacillus* metabolism during secondary metabolite production. The alkaline drift typically accompanies active production of extracellular enzymes and bioactive compounds, resulting from amino acid metabolism and ammonia release (40–42). The observed pH increase is consistent with previous reports showing that *B. subtilis* and related species exhibit enhanced secondary metabolite synthesis under slightly alkaline fermentation conditions (41, 43, 44).

### Secondary screening: divergence between agar-based and liquid-phase activity

**Fig 5:**
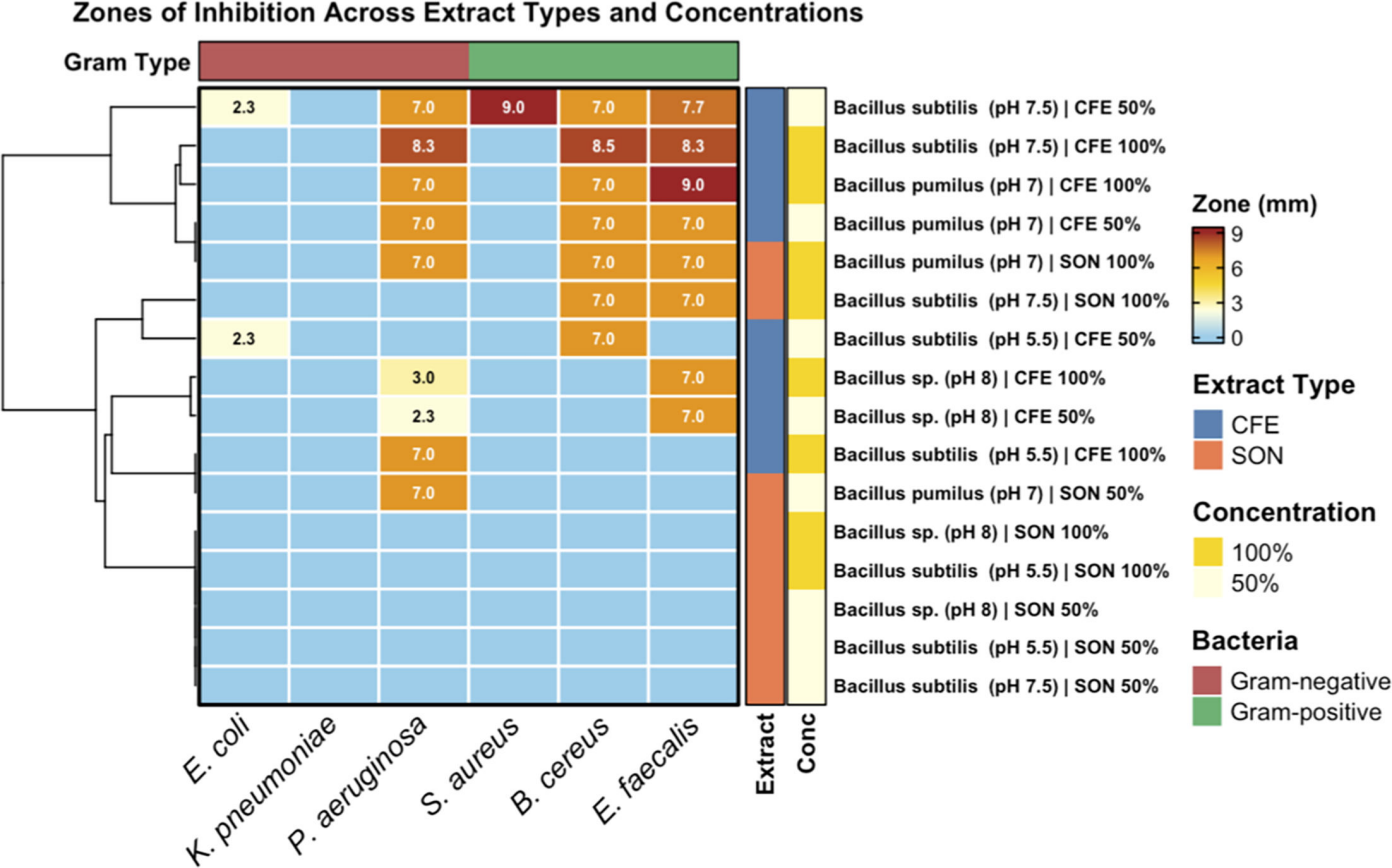
Secondary screening of cell-free and sonicated extracts against bacterial pathogens.

**Fig 6:**
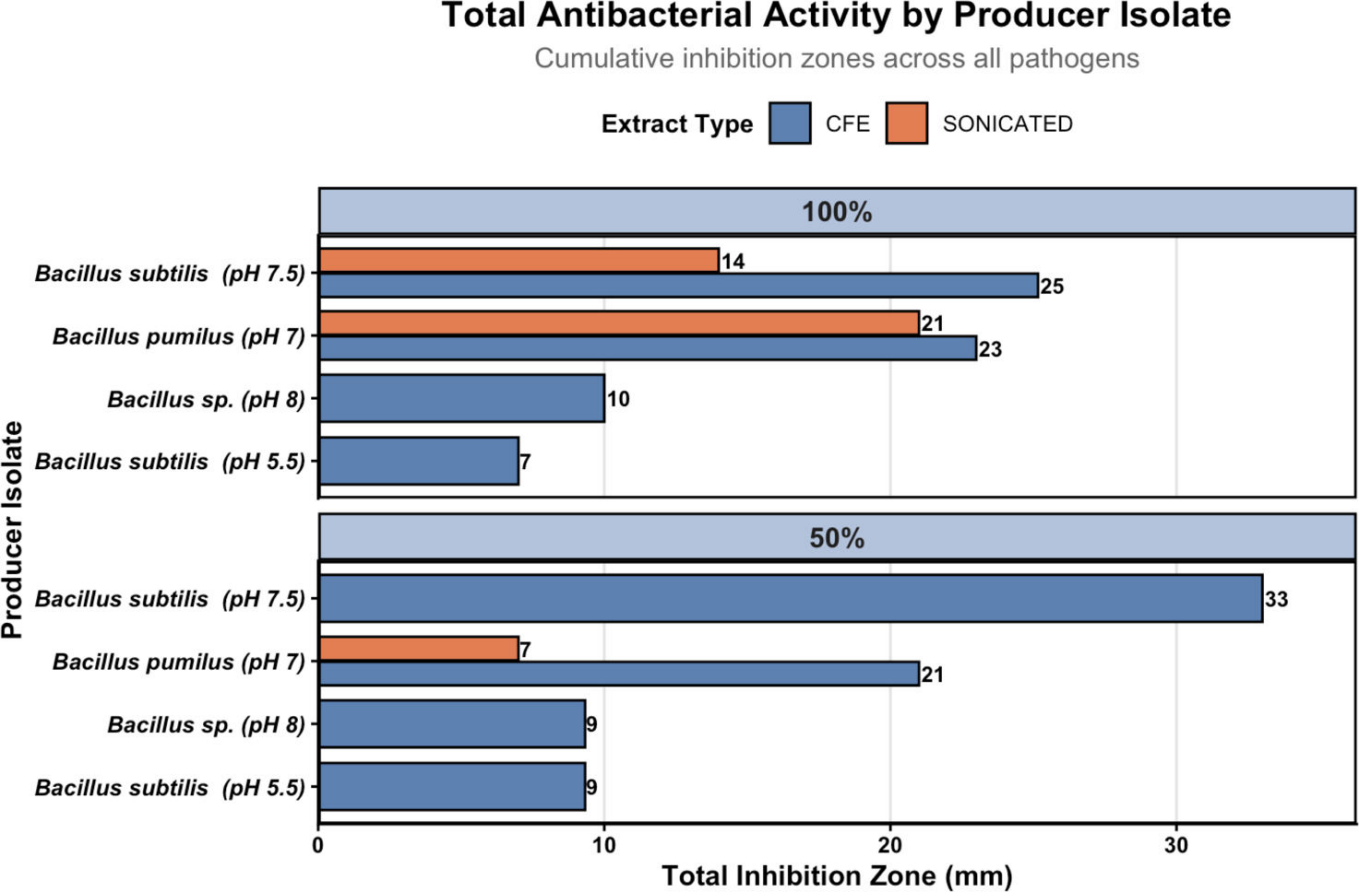
Cumulative antibacterial activity of the extracts from the four *Bacillus* producers.

Secondary screening of CFE and SON fractions using disc diffusion revealed markedly reduced antibacterial activity compared to primary agar plug assays (Fig. 5). Inhibition zones ranged from 0 to 9.0 mm, substantially lower than the 10−47.5 mm range observed during primary screening. The strongest activity (9.0 mm) was observed for putative *B. subtilis* (pH 7.5) CFE at 50% dilution, followed by moderate inhibition from *B. pumilus* (pH 7.0) extracts (7.0−8.5 mm). In contrast, only one sonicated extract showed measurable inhibition, suggesting that most bioactive metabolites are secreted extracellularly rather than retained intracellularly (Fig. 6).

### Decoupling of primary activity and secondary metabolite stability

The reduction in inhibition zones observed during secondary screening, compared to primary screening, may reflect multiple factors: (i) methodological differences between agar plug (solid-state, actively growing cells) and disc diffusion (liquid-state, cell-free extracts) assays, (ii) pH-dependent metabolite instability or degradation during fermentation, particularly under alkaline conditions (pH > 8.0), and (iii) possible shifts in BGC expression in response to pH drift. Elevated pH during fermentation may contribute to partial degradation or ionization changes in peptide-based antimicrobials, thereby reducing their diffusion in agar assays and apparent inhibitory diameter (40, 42, 44). The sustained activity observed exclusively in the pH 7.5 culture, where pH remained stable throughout fermentation, suggests that pH stability, rather than the initial pH value alone, is critical for maintaining bioactive metabolite production during submerged cultivation. This finding has important implications for fermentation process optimization in marine bioprospecting.

### Chemical diversity profiling

GC-MS analysis of ethyl acetate extracts from pH 7.0 and pH 7.5 cultures revealed the presence of over 100 putative compounds at each pH condition (7.0 and 7.5), spanning aliphatic hydrocarbons, fatty acid derivatives, alcohols, esters, and aromatic compounds. The complete list of compounds, including retention times, molecular ions, match scores, and relative abundances, is provided in Tables S1 and S2. Some of the identified compounds have been previously reported in the literature to possess antibacterial activity. Among the compounds identified were pyrrolo-[1,2-a]pyrazine-1,4-dione derivatives and cyclo-(L-prolyl-L-valine), which have been shown to exhibit antibacterial activity against human enteric and foodborne pathogenic bacteria (45, 46). Cyclo-(L-prolyl-L-valine), a diketopiperazine and cyclic dipeptide, is known to exhibit moderate antibacterial activity against a range of gram-positive and gram-negative bacteria by interfering with cell wall synthesis and quorum-sensing systems (47, 48). Similarly, pyrrolo-[1,2-a]-pyrazine-1,4-dione has been reported to inhibit both *S. aureus* and *E. coli* and to exhibit antibiofilm properties (49, 50). These compounds are frequently identified as bioactive metabolites from marine *Bacillus* species (49, 50). Full GC-MS data are provided in the supplemental material. The complete list of compounds detected in the pH 7.0 extract by GC–MS, including retention time, molecular formula, molecular weight, match score, and putative compound identity, is shown in Table S1, while Table S2 contains a list of compounds detected in the pH 7.5 extract.

Total ion chromatogram (TIC) of crude extract obtained from fermentation broth of *Bacillus* sp. at pH 7.0, analyzed by GC, is shown in Fig. S1. The chromatogram shows the overall metabolite profile, with multiple resolved peaks representing volatile and semi-volatile compounds detected across the acquisition time. Meanwhile, Fig. S2 and S3 respectively represent the TIC of crude extract obtained from fermentation broth of *Bacillus* sp. at pH 7.5, analyzed by GC–MS; and representative mass spectrum of a detected compound at retention time 20.96 min, tentatively identified as 1-docosene based on comparison with the NIST mass spectral library. Major fragment ions and matching patterns support putative identification.

Our study demonstrates that pH stability during fermentation, rather than initial cultivation pH alone, is a critical determinant of sustained antibacterial metabolite production in marine sediment *Bacillus* species. While primary agar-based screening identified multiple isolates with potent activity across a pH range of 5.0–8.0, only isolates maintained at a stable near-neutral pH (7.5) during submerged fermentation retained significant antibacterial activity in secondary liquid-phase assays. The relationship between pH and bacterial metabolism is central to the production of secondary metabolites. Optimal metabolite production generally occurs at near-neutral pH (6.5–7.5), as extreme acidity or alkalinity inhibits enzyme activity and nutrient availability for microbial growth (33). For example, studies have shown that *Streptomyces sannanensis* exhibited maximum antimicrobial production at pH 7, with minimal or no growth detected at pH 9. Similarly, *Nocardiopsis* spp. and *Streptomyces* isolates produced their highest yields at pH 7.0–8.0 (33, 34, 51). Moreover, it has been suggested that deviations from the neutral or physiological pH window can alter community composition and suppress biochemical activity (52, 53). The predominance of *Bacillus* species among culturable metabolite producers is consistent with their ecological significance in marine ecosystems (35, 37, 54, 55). The putatively identified species are well-documented producers of lipopeptides (surfactin, iturin, and fengycin), polyketides (difficidin and macrolactin), and bacteriocins, many of which exhibit broad-spectrum activity (8, 13, 37, 56). The higher inhibition zones obtained with cell-free extracts support the notion that *Bacillus* species primarily secrete their bioactive compounds into the surrounding medium rather than retaining them intracellularly, as confirmed by previous studies (57–60).

### Study limitations

Our study has several limitations, which include the pH not being monitored or controlled during fermentation, limiting the mechanistic interpretation of activity loss. Future studies should employ pH-stat fermentation or buffered media. Second, the cell-free extracts were not pH-adjusted prior to secondary screening, implying that the observed activity differences may reflect both biological and chemical factors. Third, the taxonomic identifications are putative and based solely on biochemical characterization, as molecular confirmation via 16S rRNA gene sequencing would be required for definitive species assignments. Finally, the GC-MS provided chemical diversity profiles but not bioactivity assignments, as individual compounds were not purified or bio-assayed.

### Conclusion

This study demonstrates that pH stability during fermentation is a critical determinant of sustained antibacterial metabolite production in marine sediment *Bacillus* species. While agar-based primary screening identified potent activity across a broad pH range, only cultures that maintained a stable near-neutral pH during fermentation retained bioactivity in secondary assays. These findings reveal a disconnect between agar-based screening outcomes and liquid-phase fermentation performance, emphasizing the importance of pH monitoring and control in marine bioprospecting workflows.

## Data Availability

The complete GC–MS compound identification tables and chromatograms for metabolites produced at pH 7.0 and pH 7.5 have been deposited in Figshare and are publicly available at https://doi.org/10.6084/m9.figshare.30978541.

## SUPPLEMENTAL MATERIAL

Supplemental figures aem.02595-25-s0001.docx Fig. S1 to S4.

Table S1 aem.02595-25-s0002.xlsx Peaks obtained for compounds tentatively identified in ethyl acetate extract of pH 7.0 using GC-MS.

Table S2 aem.02595-25-s0003.xlsx Peaks obtained for compounds tentatively identified in ethyl acetate extract of pH 7.5 using GC-MS.

ASM does not own the copyrights to Supplemental Material that may be linked to, or accessed through, an article. The authors have granted ASM a non-exclusive, world-wide license to publish the Supplemental Material files. Please contact the corresponding author directly for reuse.
