# Supplementary material for: pH stability during fermentation is associated with sustained antibacterial metabolite production in marine sediment Bacillus species
Source: Appl Environ Microbiol. 2026 Mar 18;92(4):e02595-25. doi: 10.1128/aem.02595-25 (PMC13101522; doi:10.1128/aem.02595-25)

**SUPPLEMENTARY FIGURES**


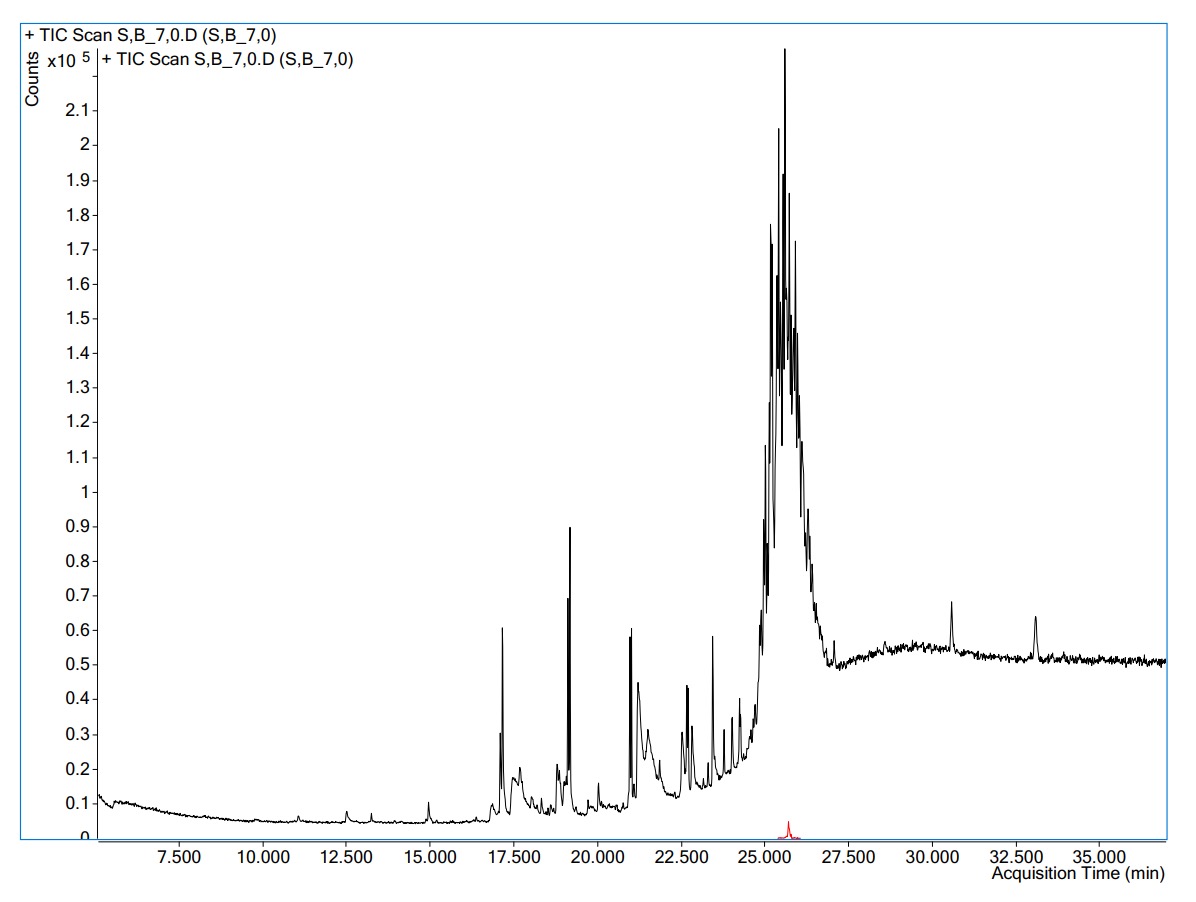


**Supplementary Figure S1.**
**Total ion chromatogram (TIC) of ethyl acetate extract of sample S_B_7.0D obtained by GC–MS analysis.**
The TIC represents the summed ion intensities across the acquisition time, illustrating the metabolic profile of the extract under identical GC–MS conditions.


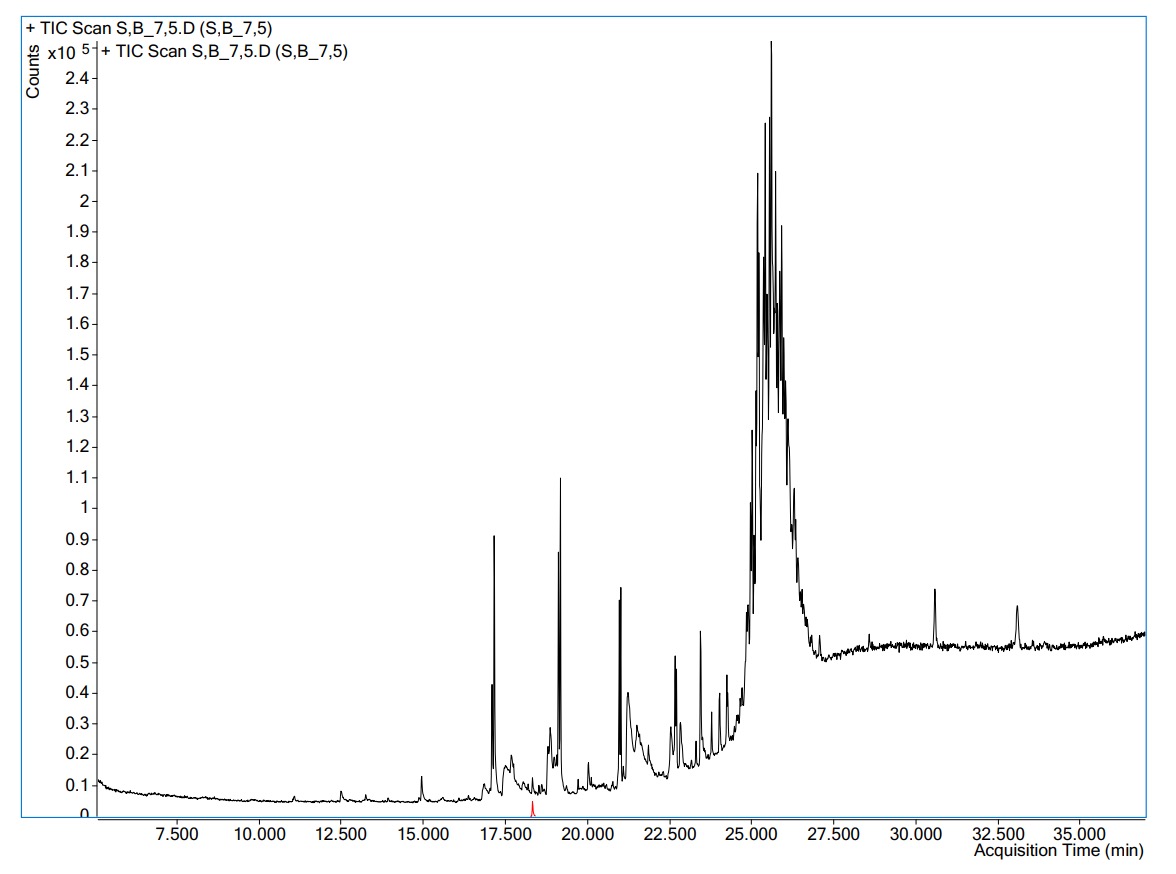


**Supplementary Figure S2.**
**Total ion chromatogram (TIC) of ethyl acetate extract of sample S_B_7.5D obtained by GC–MS analysis.**
The chromatogram shows the overall ion abundance as a function of retention time, highlighting major and minor peaks corresponding to volatile and semi-volatile metabolites detected in the extract.


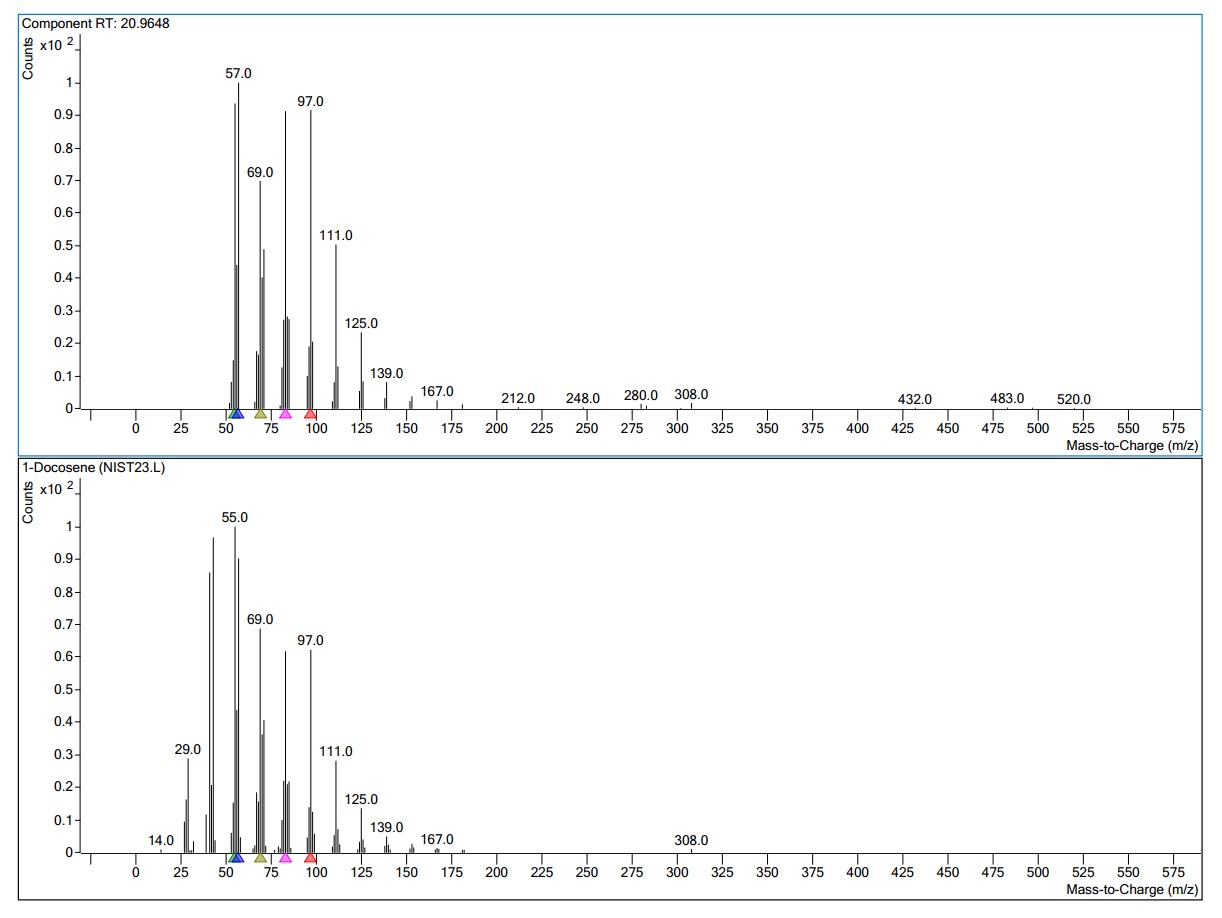


**Supplementary Figure S3**

**GC–MS mass spectral comparison of a representative compound detected at retention time (RT) 20.96 min.**
The upper panel shows the experimental electron ionization (EI) mass spectrum of the detected compound, while the lower panel displays the corresponding reference spectrum retrieved from the NIST library (1-Docosene). Key fragment ions and matching patterns support putative compound identification.

**Supplementary Figure S4: Annotation of Secondary Metabolites Using NIST Library**

The tentative identification of compounds in both sample SB 7.0 and SB 7.5 was performed using NIST library search database to ascertain the compound identity. Below is an example of a zoomed region of chromatogram (**A**) for compound 1,2-octadecanediol in sample SB 7.5. To identify the compound, the deconvoluted spectrum (**B**) is used to match the molecular ions collected to the library spectrum, resulting in accurate identification using the library match factor. Therefore, this facilitates the identification of unknown compounds using fragmentation patterns without reference standards.


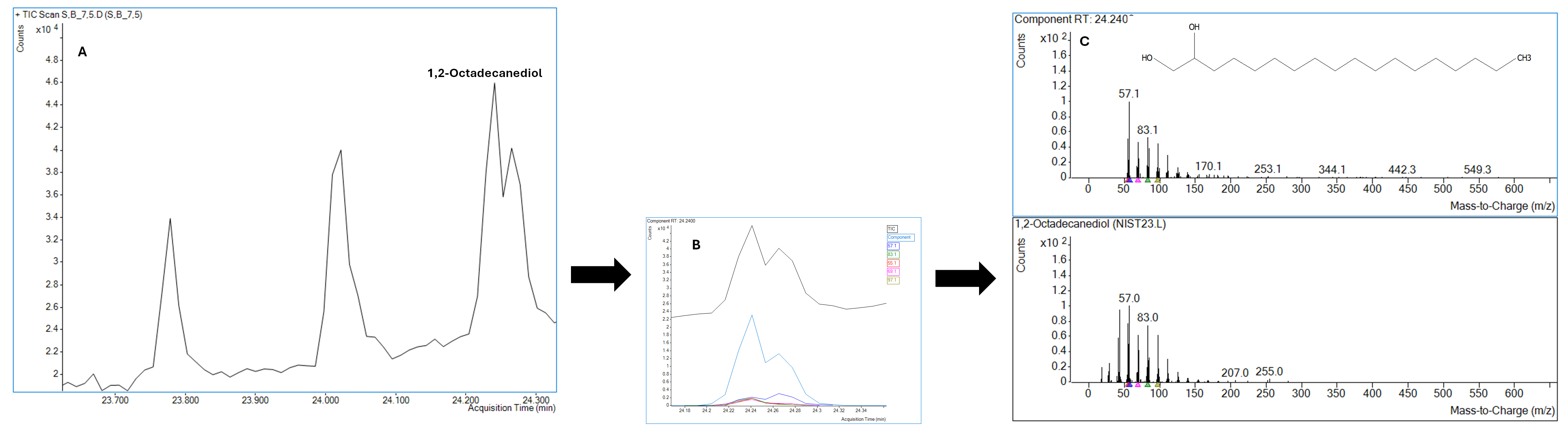


**Fig 1S: A).** a zoomed region of chromatogram or compound 1,2-octadecanediol in sample SB 7.5. **B**). The deconvoluted spectrum of 1,2-octadecanediol. **C**) acquired mass spectrum of 1,2-octadecanediol and a library spectrum.

**Same examples of Mass spectrum obtained from sample SB (pH 7.5)**


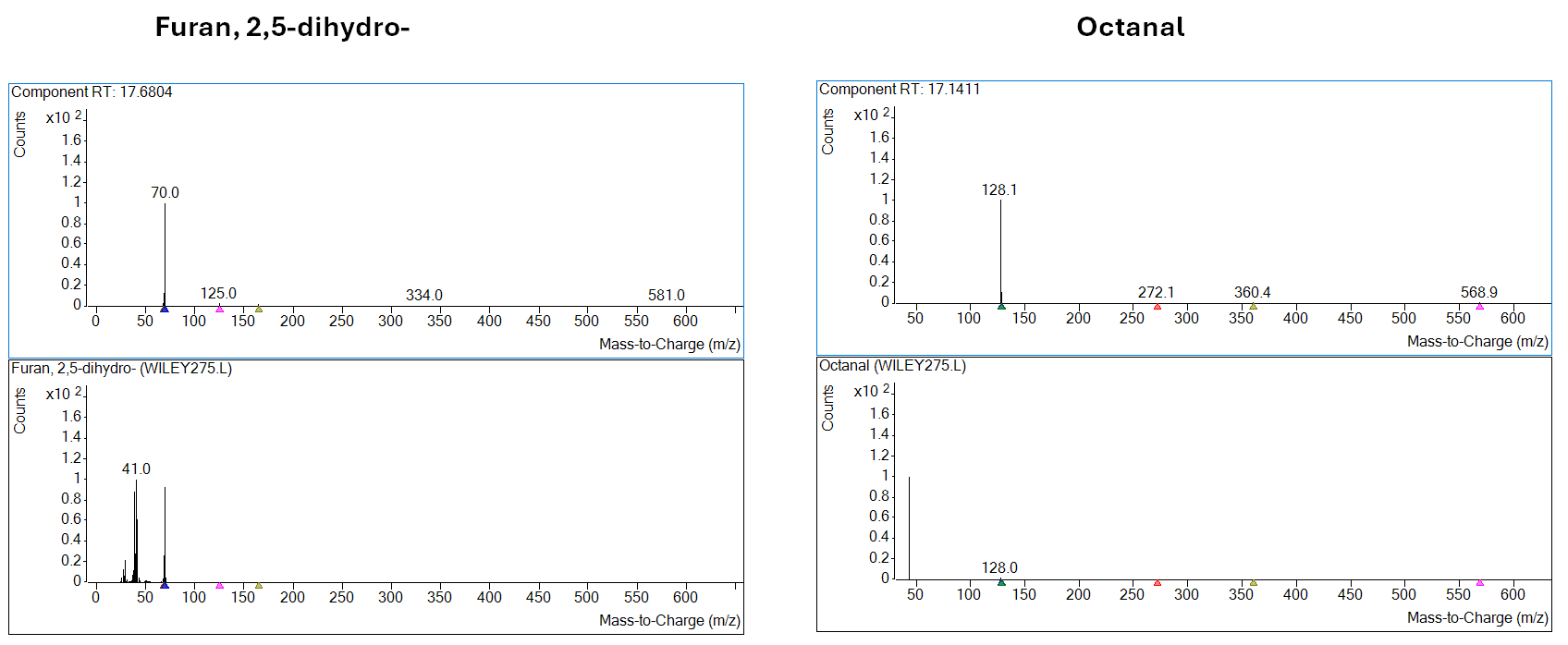


**Same examples of Mass spectrum obtained from sample SB (pH 7.0)**


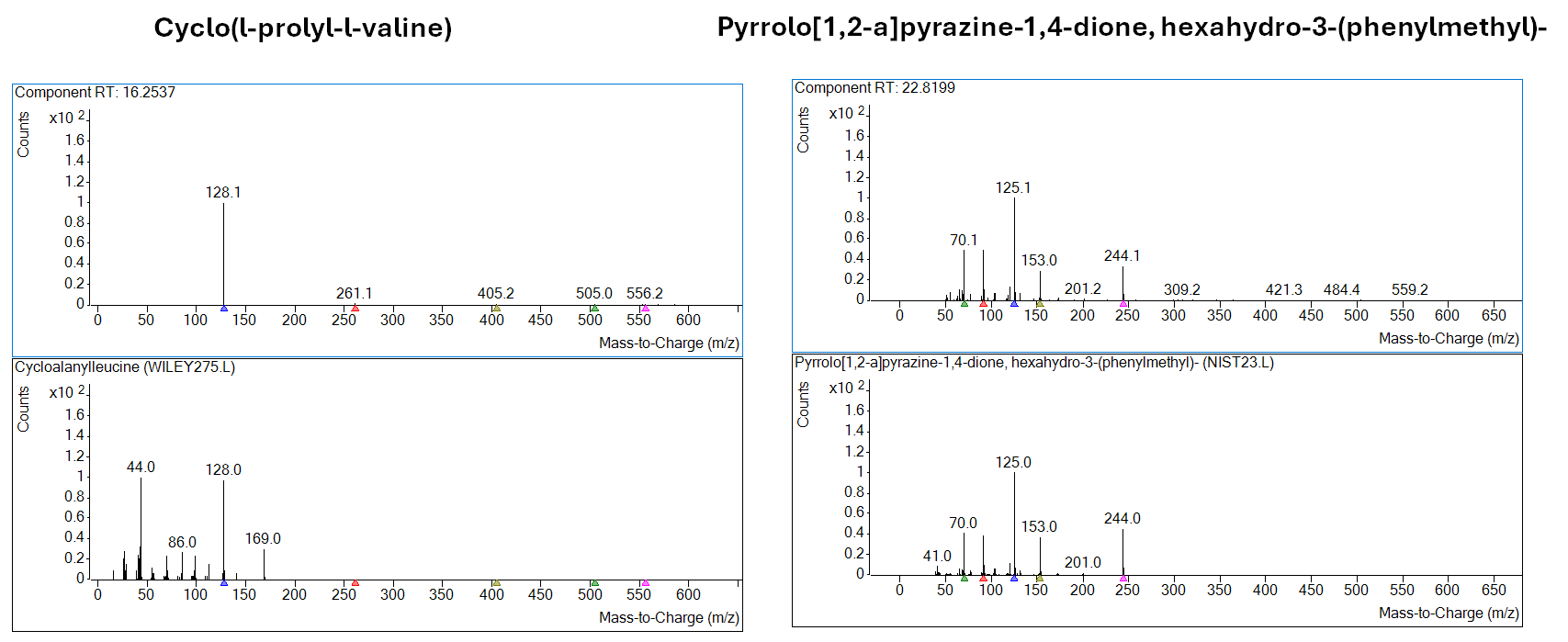


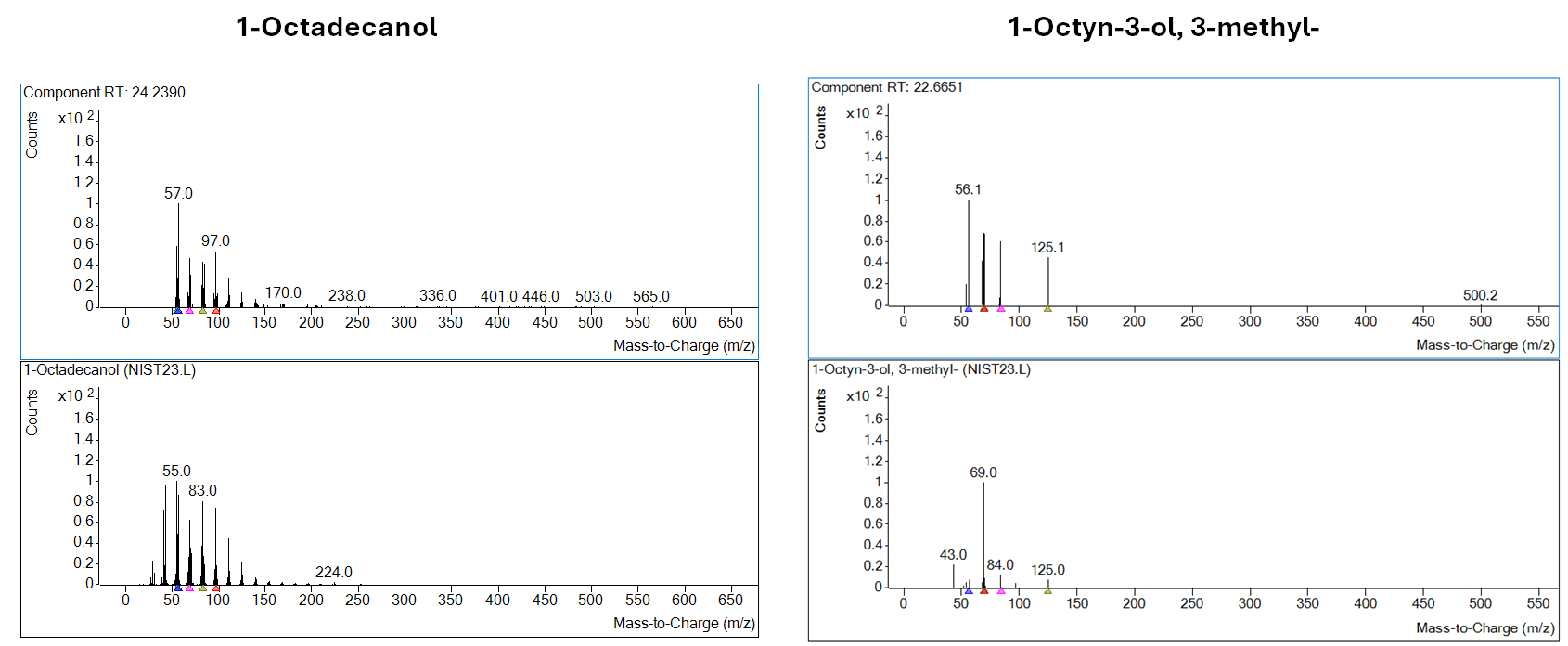


**Same examples of Mass spectrum obtained from sample SB (pH 7.5)**


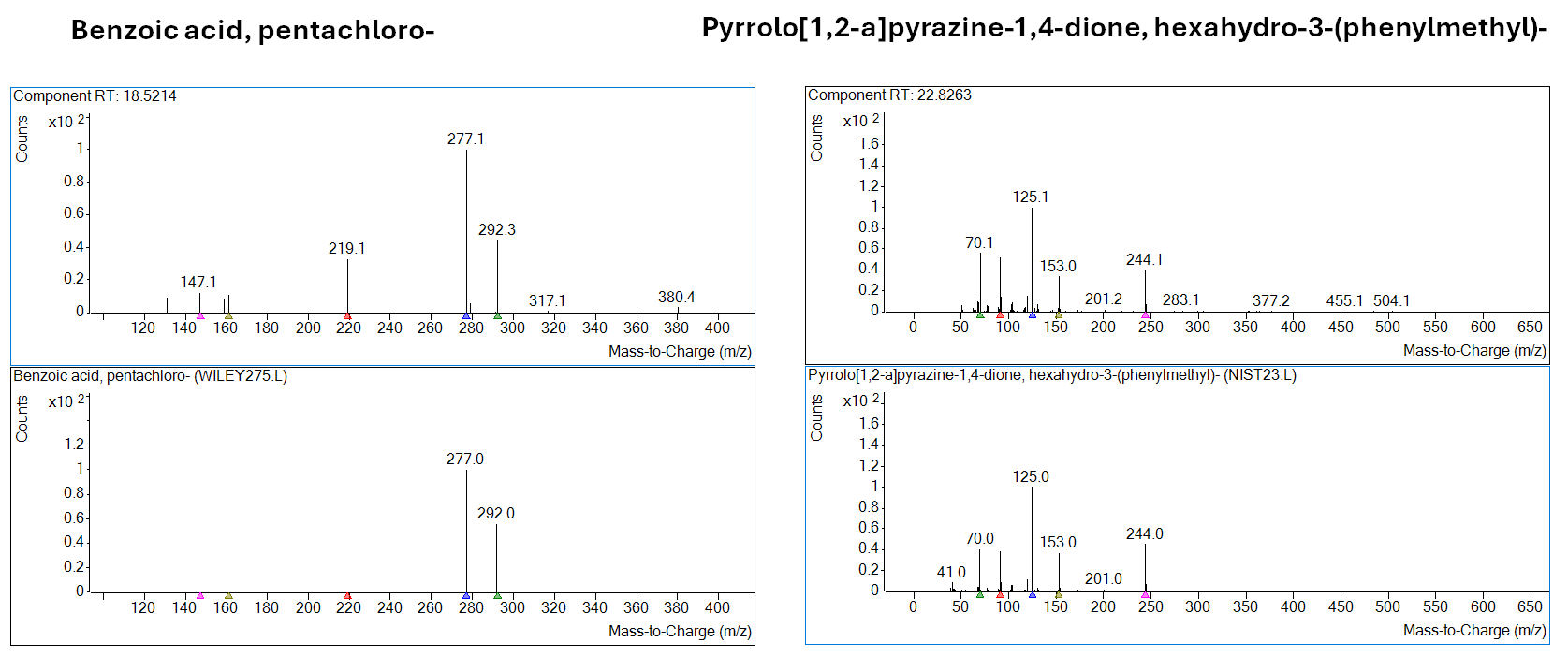


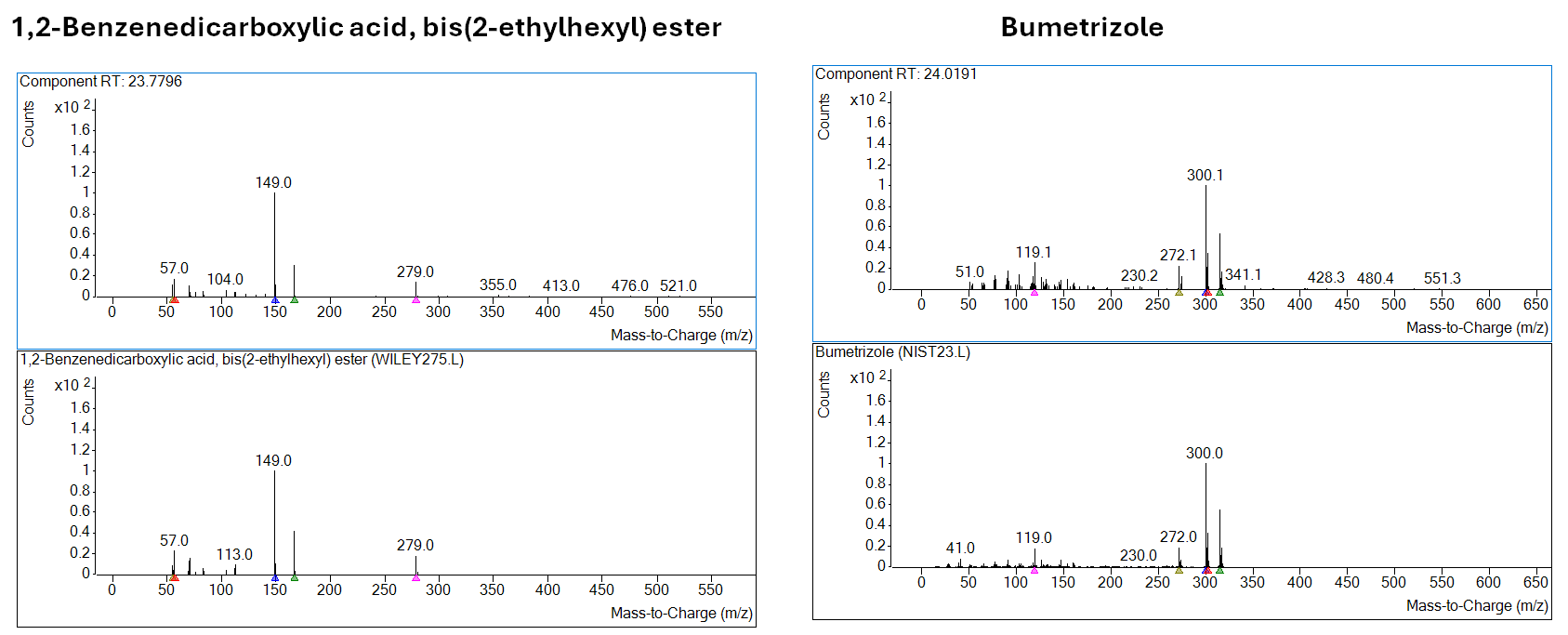

Supplement: Supplemental figures — Fig. S1 to S4. [file aem.02595-25-s0001.docx]
